# Transcriptomic profiling of microbe–microbe interactions reveals the specific response of the biocontrol strain *P. fluorescens* In5 to the phytopathogen *Rhizoctonia solani*

**DOI:** 10.1186/s13104-017-2704-8

**Published:** 2017-08-10

**Authors:** Rosanna C. Hennessy, Mikkel A. Glaring, Stefan Olsson, Peter Stougaard

**Affiliations:** 1 Department of Plant and Environmental Sciences, University of Copenhagen, Thorvaldsensvej 40, 1871 Frederiksberg C, Denmark; 2 State Key Laboratory of Ecological Pest Control for Fujian and Taiwan Crops, Fujian Agriculture and Forestry University, Fuzhou City, Fujian Province China

**Keywords:** *Pseudomonas*, Phytopathogens, Microbial interactions, Biocontrol, Transcriptomics

## Abstract

**Background:**

Few studies to date report the transcriptional response of biocontrol bacteria toward phytopathogens. In order to gain insights into the potential mechanism underlying the antagonism of the antimicrobial producing strain *P. fluorescens* In5 against the phytopathogens *Rhizoctonia solani* and *Pythium aphanidermatum*, global RNA sequencing was performed.

**Methods:**

Differential gene expression profiling of *P. fluorescens* In5 in response to either *R. solani* or *P. aphanidermatum* was investigated using transcriptome sequencing (RNA-seq). Total RNA was isolated from single bacterial cultures of *P. fluorescens* In5 or bacterial cultures in dual-culture for 48 h with each pathogen in biological triplicates. RNA-seq libraries were constructed following a default Illumina stranded RNA protocol including rRNA depletion and were sequenced 2 × 100 bases on Illumina HiSeq generating approximately 10 million reads per sample.

**Results:**

No significant changes in global gene expression were recorded during dual-culture of *P. fluorescens* In5 with any of the two pathogens but rather each pathogen appeared to induce expression of a specific set of genes. A particularly strong transcriptional response to *R. solani* was observed and notably several genes possibly associated with secondary metabolite detoxification and metabolism were highly upregulated in response to the fungus. A total of 23 genes were significantly upregulated and seven genes were significantly downregulated with at least respectively a threefold change in expression level in response to *R. solani* compared to the no fungus control. In contrast, only one gene was significantly upregulated over threefold and three transcripts were significantly downregulated over threefold in response to *P. aphanidermatum.* Genes known to be involved in synthesis of secondary metabolites, e.g. non-ribosomal synthetases and hydrogen cyanide were not differentially expressed at the time points studied.

**Conclusion:**

This study demonstrates that genes possibly involved in metabolite detoxification are highly upregulated in *P. fluorescens* In5 when co-cultured with plant pathogens and in particular the fungus *R. solani*. This highlights the importance of studying microbe–microbe interactions to gain a better understanding of how different systems function in vitro and ultimately in natural systems where biocontrol agents can be used for the sustainable management of plant diseases.

**Electronic supplementary material:**

The online version of this article (doi:10.1186/s13104-017-2704-8) contains supplementary material, which is available to authorized users.

## Background

Biocontrol bacteria provide an alternative strategy to synthetic chemicals for crop protection against disease. Many microorganisms have been shown to be effective microbial biological control agents (mBCAs) against soil-borne pathogens and in particular, fluorescent pseudomonads have been the focus of much research [12, 16, 38, 39]. Among these studies, the production of secondary metabolites and especially the role of cyclic lipopeptides in the biological control of plant pathogens has been widely documented [18, 27, 28, 30, 31].

Pseudomonads with potential applications in biocontrol have been widely studied using whole-genome sequencing in combination with functional analysis using either mutants derived by random or site-directed mutagenesis as tools for the identification of key traits underpinning biocontrol activity [4, 5, 8, 9, 24, 25, 36, 37]. However, knowledge of the overall response of biocontrol bacteria to specific pathogens is limited and therefore transcriptional profiling by RNA-seq of dual cultures is a valuable tool to elucidate potential mode of actions underpinning biocontrol bacterial antagonism toward pathogens. Despite the advances in the application of high-throughput RNA sequencing (RNA-seq) as a tool for transcriptomics, only few studies to date report using the method to study bacterial-fungal interactions [11, 26]. A recent study by [26] investigated the transcriptional response of *Serratia plymuthica* against *R. solani* in dual-culture and found that antibiosis appeared to be a key mode of action utilised by the bacterium against the pathogen.

*P. fluorescens* In5 is a potential biocontrol agent previously isolated from a disease suppressive soil in southern Greenland showing antimicrobial activity against a broad range of phytopathogens [24, 25]. Using a combination of whole-genome sequencing, mutant generation and characterisation coupled with microbial metabolomics analysis, key biocontrol traits of this isolate have recently been identified [13, 25]. In order to build upon research conducted to date and to contribute toward our knowledge of bacterial-fungal/oomycete interactions, the aim of the present study was to investigate the transcriptional changes in *P. fluorescens* In5 during dual-culture with two phytopathogens. Genome-wide RNA-seq was used as a method to investigate the transcriptional response of *P. fluorescens* In5 in dual-culture with either the basidiomycete *R. solani* or the oomycete *P. aphanidermatum* compared to a single culture of *P. fluorescens* In5 in the absence of both pathogens.

## Methods

### Dual culture assay

Nunc™ OmniTray™ (Fisher Scientific, Roskilde, Denmark) sterile plates were filled with 35 ml of fifth strength potato dextrose agar (PDA; Difco Lawrence, KS) and ten 5 mm plugs of either *R. solani* or *P. aphanidermatum* were place in the center of the plate (see Additional file 1 for plate layout) and incubated 24 h at 20 °C. *P. fluorescens* In5 was grown in 10 ml Luria–Bertani broth overnight at 28 °C and subsequently streaked 3 cm away from the fungal or oomycete plugs using a sterile inoculation loop. Plates were incubated at 20 °C for 48 h.

### RNA isolation

At 48 h, bacterial cells representing biological triplicates were scraped into 500 µl of RNAlater^®^ (Thermo Fisher), micropipetted and briefly vortexed before being stored at 4 °C overnight. Before proceeding to RNA extraction, 1 ml of cold phosphate-buffered saline (PBS) was added to each tube containing 500 µl RNAlater^®^ and centrifuged 5 min at 14,000 g and supernatant removed before proceeding to RNA extraction. Total RNA was isolated using the ZR Fungal/Bacterial RNA MiniPrep™ kit according to the manufacturer’s instructions (Zymo Research, Nordic Biosite, Copenhagen, Denmark).

### RNA sequencing

RNA sequencing (RNA seq) libraries were constructed and sequenced following a default Illumina stranded RNA protocol including rRNA depletion (BGI tech, Hong Kong, China). The short-insert library was sequenced on an Illumina HiSeq system by 2 × 100 bp paired-end sequencing producing approximately 10 million reads per sample. Trimming and quality filtering of sequences and transcriptomics analysis were performed using CLC Genomics Workbench (CLC bio, Qiagen, Aarhus, Denmark). The trimmed sequences were mapped to the annotated In5 genome (GenBank accession no. LIRD01000000) using default options for prokaryotes, except that the minimum length and similarity fraction of a matched read was set to 0.8 and 0.9, respectively. Only reads where both ends of a paired-end read could be mapped were counted, all other reads were discarded. Expression values for individual coding sequences (CDS) were calculated as Reads Per Kilobase of transcript per Million mapped reads (RPKM). The average expression value from the three biological replicates was used to calculate fold change differential expression of all annotated CDS in response to *R. solani* or *P. aphanidermatum* compared to the control (*P. fluorescens* In5 single culture). For calculating fold-change, only genes with a minimum expression value (RPKM) >5 in both control and interaction was included.

Significance of the transcriptomic dataset was calculated as follows. For the three replicate RPKM values (control, Rs, Pa) the standard error (SE) was calculated for each gene and for the three treatments (control, Rs, Pa). The standard error of the means (SEM) was subsequently calculated for the ratio of the two fungal treatments (Rs, Pa) and the control treatment (C). From the resulting values, the margin of error (MOE) was calculating containing 95% of the distribution (MOE = 1.96*SEM). Finally, for significance testing the following was determined: if treatment/control ratio − MOE is larger than 1 then fungal treatment is significantly larger (*P* < 0.05) than control. If the ratio + MOE is smaller than 1 then the fungal treatment is significantly smaller (*P* < 0.05) than control.

## Results and discussion

### Phenotypic analysis and targeted response against specific pathogens by *P. fluorescens* In5

In order to gain insight into the *P. fluorescens* In5 mechanism of action against phytopathogens, a dual-culture assay was established to study the bacterial transcriptome in response to *R. solani* and *P. aphanidermatum*. Transcriptional profiling of *P. fluorescens* In5 using RNA-seq was conducted on single bacterial cultures as a control, or in dual-culture with either *R. solani* or *P. aphanidermatum*. Approximately 10 million paired-end sequence reads were generated from each of the three biological replicates for each setup and gene expression levels were determined by comparison to the available genome sequence [13].

**Fig. 1:**
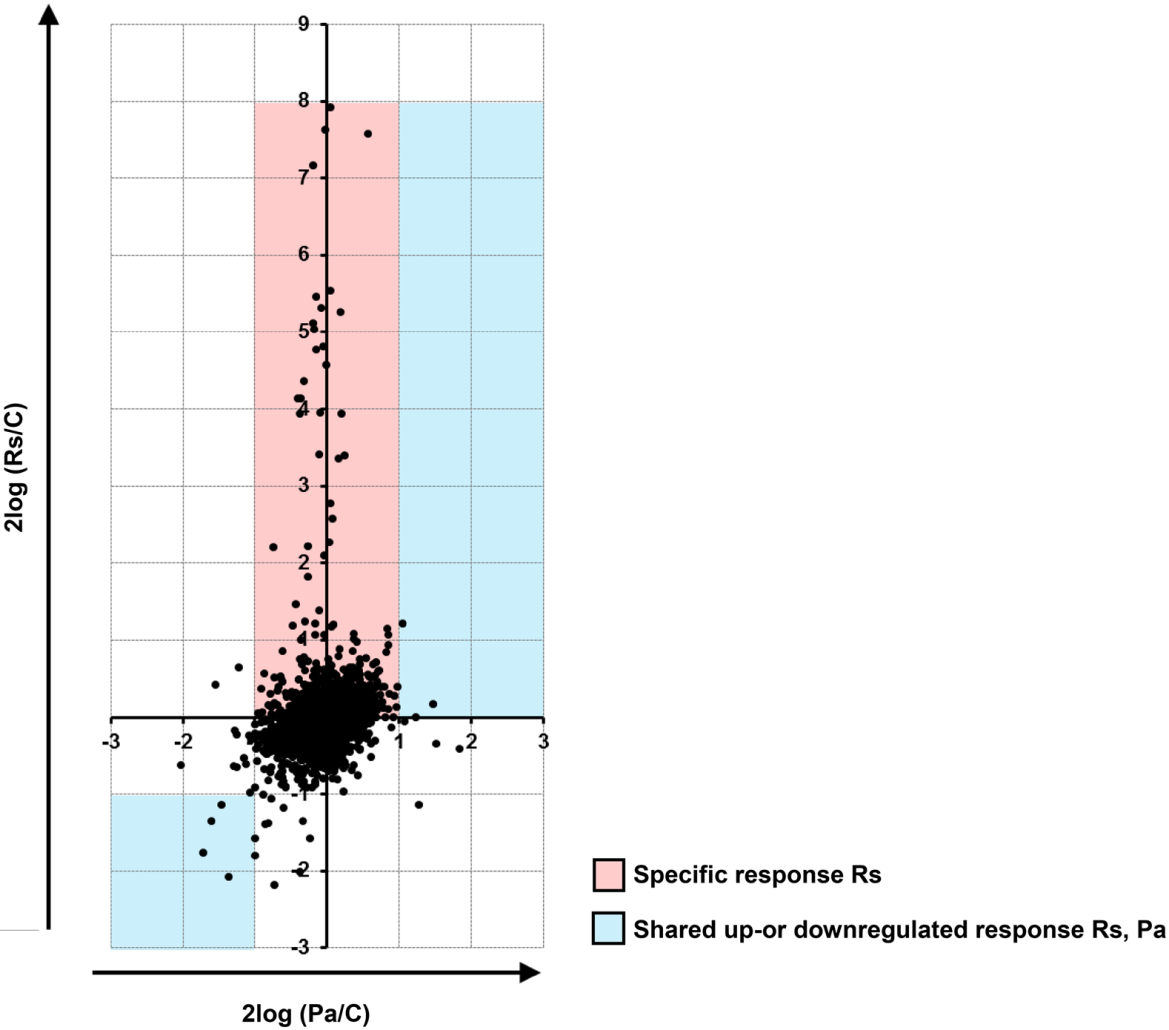
Gene expression 2log plot of *P. fluorescens* In5 during interactions with *R. solani* and *P. aphanidermatum*. Graph represents 2log of expression means for each treatment (Rs, *R. solani* or Pa, *P. aphanidermatum*) compared to the control (C)

The most striking feature of this comparative transcriptomics study was the organism-specific response of *P. fluorescens In5*. Overall, the majority of genes were unchanged in expression relative to the control (Fig. 1; Additional file 2). Importantly, only two transcripts were up-or downregulated at least twofold in response to both *R. solani* and *P. aphanidermatum* (Table 1; Additional file 3: Table S1; Table 2; Additional file 4: Table S2), an upregulated gene encoding an Mbth-like protein (AL066_04890) and a downregulated gene encoding a putative AlpA transcriptional regulator (AL066_26360). Interestingly, no transcripts were upregulated more than twofold in *R. solani* and downregulated more than twofold in *P. aphanidermatum* or vice versa (Additional file 2).

**Table 1:** Differential expression of *P. fluorescens* In5 genes during an interaction with *R. solani* compared to *P. aphanidermatum*

Locus tag	Protein name	Fold change	Fold change
		*R. solani*	*P. aphanidermatum*
AL066_06105	Alpha/beta hydrolase	239.5	1.0
AL066_09895	Hydrolase	194.8	−1.0
AL066_14420	Quercetin 2,3-dioxygenase	188.8	1.5
AL066_18305	FMN-dependent NADH-azoreductase	142.0	−1.1
AL066_05010	Hypothetical protein	45.9	1.0
AL066_10105	Pirin	43.4	−1.1
AL066_04230	Aromatic ring-opening dioxygenase LigB	39.4	−1.1
AL066_06100	Mechanosensitive ion channel protein MscS	37.8	1.1
AL066_31290	Hypothetical protein	34.2	−1.1
AL066_13550	Phage infection protein	32.4	−1.1
AL066_14055	Hypothetical protein	27.6	−1.0
AL066_13570	Hypothetical protein	27.1	−1.1
AL066_05530	Glutathionyl-hydroquinone reductase YqjG	23.5	−1.0
AL066_06700	Hypothetical protein	20.4	−1.3
AL066_06705	Hypothetical protein	17.4	−1.3
AL066_12150	Hypothetical protein	17.4	−1.3
AL066_12145	Hypothetical protein	15.3	−1.1
AL066_07630	Hypothetical protein	15.1	−1.3
AL066_07090	ATPase	15.1	1.2
AL066_13695	Hypothetical protein	10.6	−1.1
AL066_31095	Filamentous hemagglutinin	10.4	1.2
AL066_10590	FMN-dependent NADH-azoreductase	10.1	1.1
AL066_31575	Quercetin 2,3-dioxygenase	6.8	1.0
AL066_04065	DoxX family protein	5.9	1.1
AL066_07095	Histidine kinase	4.8	1.0
AL066_22770	Hypothetical protein	4.6	−1.2
AL066_12155	Hypothetical protein	4.6	−1.7
AL066_03355	ABC transporter	4.2	−1.0
AL066_27880	Cytochrome b	3.5	−1.2
AL066_11190	Oxidoreductase	−3.0	−2.0
AL066_11490	Hypothetical protein	−3.0	−1.2
AL066_26360	AlpA family transcriptional regulator	−3.5	−3.3
AL066_11195	Sulfite reductase	−3.5	−2.0
AL066_13155	NIPSNAP domain containing protein	−4.1	−1.3
AL066_11200	Cytochrome C oxidase Cbb3	−4.3	−2.6
AL066_11050	(Fe–S)-binding protein	−4.6	−1.7

Values indicate fold change based on mean expression values across biological triplicates compared to the control (bacteria only). Only genes showing at least a threefold change in response to *R. solani* are shown. The protein names are derived from the automated GenBank annotation of the genome

**Table 2:** Differential expression of *P. fluorescens* In5 genes during an interaction with *P. aphanidermatum* compared to *R. solani*

Locus tag	Protein name	Fold change	Fold change
		*P. aphanidermatum*	*R. solani*
AL066_27440	Hypothetical protein	3.6	−1.4
AL066_27625	Glycine/betaine ABC transporter substrate-binding protein	2.9	−1.3
AL066_02610	Hypothetical protein	2.8	1.1
AL066_27960	Malonate decarboxylase subunit delta	2.4	−2.2
AL066_25950	Hypothetical protein	2.4	−1.0
AL066_07605	ABC transporter permease	2.1	−1.1
AL066_04890	Antibiotic synthesis protein MbtH	2.1	2.3
AL066_08545	PseC, RND transporter	−2.1	−1.2
AL066_05775	Hypothetical protein	−2.1	−2.0
AL066_27365	Biotin synthase	−2.1	−1.2
AL066_11260	Cytochrome C	−2.2	−1.5
AL066_07745	Beta-lactamase	−2.2	−1.5
AL066_17410	Hypothetical protein	−2.3	1.6
AL066_14095	Hypothetical protein	−2.4	−1.6
AL066_23365	Hypothetical protein	−2.4	−1.2
AL066_17940	Phenylalanine 4-monooxygenase	−2.4	−1.2
AL066_10185	Hypothetical protein	−2.4	−1.6
AL066_11200	Cytochrome C oxidase Cbb3	−2.6	−4.3
AL066_15755	Terminase	−2.8	−2.2
AL066_15780	Transcriptional regulator	−2.9	1.3
AL066_24330	Hypothetical protein	−3.0	−2.6
AL066_26360	AlpA family transcriptional regulator	−3.3	−3.5
AL066_11985	Hypothetical protein	−4.1	−1.6

Values indicate fold change based on mean expression values across biological triplicates compared to the control (bacteria only). Only genes showing at least a twofold change in response to *P. aphanidermatum* are shown. The protein names are derived from the automated GenBank annotation of the genome

During dual cultivation of the bacterium with *R. solani*, a zone of inhibition was established between *P. fluorescens* In5 and the fungus which persisted for over three weeks. Analysis of dual-cultures of *P. fluorescens* In5 and *R. solani* identified a total of 37 genes, including 12 hypothetical genes, with at least a threefold change in expression level compared to the control (Table 1). Of the 37 differentially expressed transcripts, 30 were shown to be significant (Additional file 3: Table S1). In contrast, only four genes showed significant differential gene expression by more than threefold change during the interaction with *P. aphanidermatum* (Table 2; Additional file 4: Table S2). Among the 37 differentially expressed genes during the interaction with *R. solani*, 29 genes were upregulated and eight genes were downregulated.

### Upregulation of genes involved in secondary metabolism and detoxification in response to *R. solani*

Many of the genes of known function whose expression was highly upregulated in response to *R. solani* encode enzymes associated with aromatic compound metabolism and detoxification (Table 1; Additional file 3: Table S1). Two genes, both putatively coding for quercetin 2,3-dioxygenases (AL066_14420, AL066_31575) were upregulated over sixfold. In the rhizosphere, microbes are continually exposed to secondary metabolites including aromatic compounds and flavonoids such as quercetin, which has been shown to possess antibacterial activity by inhibiting DNA gyrase [17, 29]. Quercetin 2, 3-dioxygenase is required for quercetin degradation and functions by opening the C-ring forming a depside and releasing a carbon monoxide. In addition to the upregulation of quercetinases, two hydrolases (AL066_06105, AL066_09895) were the most highly upregulated genes in response to *R. solani*. The first hydrolase (AL066_06105) shows 83% identity to a characterised esterase from *P. putida*, while the second hydrolase (AL066_09895) is distantly related by 31% to a phosphoesterase from *E. coli.* The final step in quercetin metabolism is hydrolysis of the depside formed from the dioxygenase activity by an esterase to yield 2-protocate-chuoyl-phloroglucinol carboxylic acid and protocatechuic acid [15]. In relation to the rhizosphere, it has been reported that the ability of the biocontrol strain *P. fluorescens* WCS365 to utilise organic acids is the nutritional basis for tomato root colonisation by the bacterium [23]. These results suggest that *P. fluorescens* In5 detoxifies fungal-derived aromatic compounds potentially produced by *R. solani* in response to the bacterial interaction and possibly also subsequently metabolises the degradation products. To the best of our knowledge, *R. solani* or other fungi have not yet been shown to naturally produce quercetin with the exception of a medicinal plant endophyte [22]. However, quercetin 2,3-dioxygenases have been shown to act on different flavonoids indicating the potential production of phenolic-like compounds by *R. solani*, which has been documented for *Rhizoctonia* spp. [6, 21, 33]. Additional significantly upregulated genes relating to aromatic compound metabolism were two FMN-dependent NADH-azoreductases (AL066_18305, AL066_ 10590), an aromatic ring-opening dioxygenase (AL066_04230) and a glutathionyl-hydroquinone reductase (AL066_05530).

Analysis of the quercetinases discussed above found that both proteins belong to the pirin family with one of the enzymes (AL066_31575) possessing the pirin c-terminal cupin domain. In addition to these two enzymes, a pirin-like protein encoding gene (AL066_10105) was also significantly upregulated (>40-fold change) in response to *R. solani*. In *P. stutzeri*, a pirin-like protein has been shown to possess quercetinase activity, although the biological function of pirin remains largely unknown [1]. In eukaryotes, pirin has been proposed to be involved in transcriptional activation and cell apoptosis, while in prokaryotes, for example in cyanobacteria, it has been shown to be stress induced [1, 14, 34, 40].

### Interaction with *R. solani* induces the expression of small hypothetical proteins

A notable feature of the transcriptional response of *P. fluorescens* In5 to *R. solani* was the upregulation of several genes encoding hypothetical proteins (Table 1; Additional file 3: Table S1). The genes were found to encode small proteins all predicted to be secreted with the exception of AL066_12150 (Additional file 5). Antimicrobial peptides (AMPs) are small molecules produced and secreted by diverse organisms and can be referred to as cationic host defence peptides, anionic or cationic peptides or alpha-helical antimicrobial peptides among others [2]. Antifungal peptides can be diverse in structure and typically target fungal cell walls or membranes; for example they can bind the major structural component of fungal cells walls chitin, or disrupt fungal membranes increasing permeability or directly form pores [2]. These hypothetical proteins could be involved in the response of the bacterium to *R. solani* or antagonism toward the fungus. However, further studies providing proteomic or immunological evidence is required to establish whether these proteins are present extracellularly.

In contrast to *R. solani*, there was no significant inhibition zone observed for the interaction between *P. fluorescens* In5 and *P. aphanidermatum* at 48 h, though the oomycete did not grow past the bacterium. The anti-*Pythium* activity did not however persist beyond 72 h dual cultivation. In response to *P. aphanidermatum*, there were no genes showing threefold or greater differential expression relative to the control (Table 2). Of the genes recorded to have fold-change values above two, nine transcripts were upregulated and 16 downregulated. Among the upregulated genes, five encode hypothetical proteins and the remaining genes putatively encode three transporters (AL066_27625, AL066_07870, AL066_7605), a malonate decarboxylase delta subunit (AL066_27960) and an antibiotic synthesis mbth protein (AL066_04890). These results indicate that antibiosis may be a mode of action by *P. fluorescens* In5 during the interaction with *P. aphanidermatum* similarly to the mechanism reported to underpin the interaction between *S. plymuthica* and *R. solani* [26]. Transcripts downregulated by a fold-change greater than two included two hypothetical genes (AL066_11985, AL066_24330) in addition to a putative AlpA transcriptional regulator (AL066_26360) which as mentioned previously, was also downregulated during the *R. solani* interaction.

### Interaction with *P. aphanidermatum* induces an Mbth-like protein and ABC-transporter like encoding genes

As discussed earlier, differential expression analysis showed few to no changes in response to the oomycete *P. aphanidermatu*m. Among the *Pythium*-induced genes, two ABC-transporter like genes were strongly upregulated (AL066_27625, AL066_07605). Biocontrol agents must be able to tolerate antibiotics produced by plant pathogens in addition to their own compounds and thus upregulation of these genes could indicate a potential role in cell detoxification during the biocontrol interaction [32]. Interestingly, a beta-lactamase gene (AL066_07745) typically required for antibiotic resistance against beta-lactams was downregulated. This could point to *Pythium* not producing beta-lactams in response to In5 or that In5 responds specifically to fungi rather than oomycetes, which more closely resemble plants. The latter is in accordance with the transcriptomic data here presented whereby the *Pythium* interaction when compared to the control showed few to no up-or-downregulated transcripts.

Another upregulated transcript was an MbtH-like protein (AL066_04890) located downstream of an NRPS encoding gene (AL066_04845). MbtH-like proteins are small proteins of unknown function although a potential role in NPRS biosynthesis has been proposed [3, 10]. It has also been reported that co-production of such proteins with NPRS components can enhance protein production [10]. Interestingly, MbtH-like proteins have been proposed as useful target genes for the identification of novel secondary metabolite gene clusters by genome mining [3]. Genome analysis of In5 identified a second MbtH-like protein also located downstream of an NRPS gene cluster. Of note, disruption of this cluster renders the mutant unable to inhibit *Pythium* and switches off nunapeptin production (data not shown).

### Identification of co-expressed gene clusters during bacterial-phytopathogen interactions

**Fig. 2:**
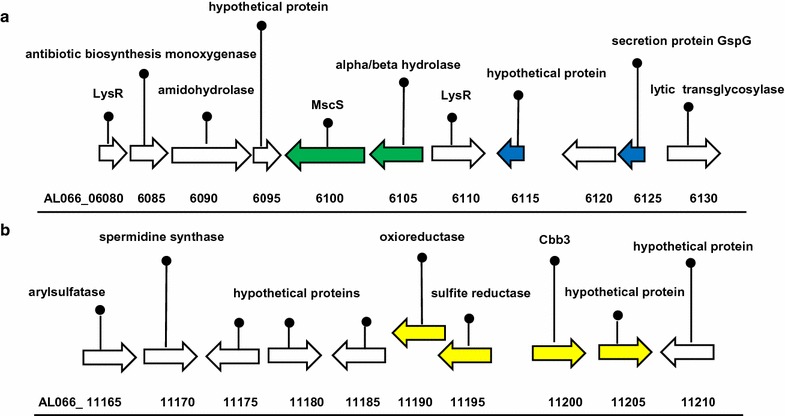
Example of the clustering of genes differentially regulated in response to both *R. solani* and *P. aphanidermatum* (**a**) or *R. solani* (**b**). Genes upregulated in response to *R. solani* are represented by green arrows, genes upregulated in response to both *R. solani* and *P. aphanidermatum* are represented by *blue arrows*, and *yellow arrows* indicate genes downregulated in response to *R. solani*

The transcriptomic study conducted by [26] reported the upregulation of genes in clusters. Clustering of differentially expressed genes was also observed in this study for a number of transcripts in response to both phytopathogens (Fig. 2). For example, four genes in a cluster were upregulated in response to *R. solani* or both *R. solani* and *P. aphanidermatum* (Fig. 2a). Interestingly, within this cluster, two putative LysR transcriptional regulators (AL066_06080, AL06110) were identified. Although not differentially expressed at 48 h, these regulator-like proteins may play an important role in antifungal activity at earlier or later time points. In *P. chlororaphis*, a knockout mutant of *ptrA* encoding a LysR-type regulator was shown to be defective in antifungal activity against *Scelorotinia sclerotiorum*, indicating a functional role relating to the biocontrol activity of the bacterium [20]. Four of the downregulated genes in *P. fluorescens* In5 in response to *R. solani* were clustered and putatively encode proteins involved in sulphur oxidation (Fig. 2b). Expression of these genes might be more relevant in response to the microbial production of sulfur-containing compounds or environments rich in organic C such as the rhizosphere [7, 19, 35]. At present, the role of genes located up- or downstream of differentially expressed transcripts remains to be determined.

In a previous study, the non-ribosomal peptides (NRPs) nunamycin and nunapeptin were characterised as key components of the biocontrol activity of *P. fluorescens In5* [25]. None of the genes with the exception of *pseC*, located on the NRPs-encoding genomic island were differentially regulated in response to *R. solani* or *P. aphanidermatum* at the time point tested. As production of the peptides decreases after 48 h (data not shown), it is likely that the biosynthesis genes are upregulated in the early stages of the interaction and once the non-ribosomal peptides synthetase (NRPS) genes are expressed, the mega-enzyme complex is formed and directs peptide biosynthesis independently from ribosomes and mRNA. An important limitation of this study was the technical issue of the quantity of RNA that could be extracted at early time-points and consequently 48 h was selected for analysis similarly to the study described by [26]. Overall, the results from this study investigating the transcriptional response of *P. fluorescens* In5 during interactions with two phytopathogens, point towards a general mechanism of antibiotic biosynthesis and transport and most notably an organism-specific mode of action which in response to *R. solani* is secondary metabolite detoxification, degradation and metabolism.

## Conclusions

Transcriptomic profiling of the *P. fluorescens* In5 responses toward two significant phytopathogens has directed our genomic analysis of biocontrol traits to reveal previously uncharacterised genes for future functional genomics studies. In addition to genes of known function, many genes encoding proteins of unknown function were differentially expressed indicating that multiple genes and processes appear to be involved in both the antagonism of *P. fluorescens* In5 toward phytopathogens and the bacterium’s response. It has been widely documented that the primary mechanism of pathogen inhibition by biocontrol bacteria is the production of antimicrobial secondary metabolites. Based on the results presented here, transcripts required for secondary metabolite degradation were highly upregulated indicating that secondary metabolite detoxification may be a key defence mode of action during the interplay between *P. fluorescens* In5 and plant pathogens. This preliminary transcriptome analysis also demonstrates that *P. fluorescens* In5 is specialised in the antagonism of fungal pathogens and, while having demonstrated anti-*Pythium* activity, the response of the bacterium toward the oomycete is not as defined. Going forward it will be important to conduct similar transcriptomic-based studies. Ultimately, understanding how biocontrol agents respond to different pathogens is critical if such microbes are to play a role in the management of plant diseases.

## Additional files

**Additional file 1.** Dual-culture assay plate layout for studying bacterial-fungal or oomycete interactions. A Nunc™ OmniTray™ was prepared with 35 ml of fifth potato dextrose agar (PDA) with 10 plugs as inoculum (5 mm) of either *R. solani* or *P. aphanidermatum*. *P. fluorescens In5* cells were streaked 3 cm away from the plugs and incubated at 48 h at 20 °C.

**Additional file 2.** Gene expression matrix of RNA-seq data of *P. fluorescens* In5 during interactions with *R. solani* and *P. aphanidermatum*. Graph represents 2log of expression means for each treatment (*R. solani*, Rs or *P. aphanidermatum*, Pa) compared to the control (C).

**Additional file 3: Table S1.** Significance testing of transcriptomic data. Transcripts significantly (P<0.05) up- (↑) or downregulated (↓) from the control (*Pseudomonas fluorescens* In5) in dual-culture with *Rhizoctonia solani* (Rs) compared to *Pythium aphanidermatum* (Pa) are indicated by 1 (red box) whereas transcripts not significantly (*P>0.05*) differentially expressed from control are represented as 0 (green box). Only genes up-or downregulated three-fold were included.

**Additional file 4: Table S2.** Significance testing of transcriptomic data. Transcripts significantly (*P<0.05*) up- (↑) or downregulated (↓) from the control (*Pseudomonas fluorescens* In5) in dual-culture with *Pythium aphanidermatum* (Pa) compared to *Rhizoctonia solani* (Rs) are indicated by 1 (red box) whereas transcripts not significantly (*P>0.05*) differentially expressed from control are represented as 0 (green box). Only genes up-or downregulated two-fold were included.

**Additional file 5.** Characteristics of the hypothetical proteins encoded by genes upregulated in response to *R. solani.*

## Abbreviations

mBCAs: microbial biological control agents
NRPS: non-ribosomal peptide synthetases
RNA-seq: RNA sequencing
