# Supplementary material for: Transcriptomic profiling of microbe–microbe interactions reveals the specific response of the biocontrol strain P. fluorescens In5 to the phytopathogen Rhizoctonia solani
Source: BMC Res Notes. 2017 Aug 10;10:376. doi: 10.1186/s13104-017-2704-8 (PMC5557065; doi:10.1186/s13104-017-2704-8)
Supplement: Supplementary file 1 — Additional file 1. Dual-culture assay plate layout for studying bacterial-fungal or oomycete interactions. A Nunc™ OmniTray™ was prepared with 35 ml of fifth potato dextrose agar (PDA) with 10 plugs as inoculum (5 mm) of either R. solani or P. aphanidermatum. P. fluorescens In5 cells were streaked 3 cm away from the plugs and incubated at 48 h at 20 °C. [file 13104_2017_2704_MOESM1_ESM.docx]

**3 cm**

**5 mm plugs inoculum**

***R. solani* or *P. aphanidermatum***

***P. fluorescens* In5**

**Growth after 24 hours**

**Additional File 1.** Dual-culture assay plate layout for studying bacterial – fungal or oomycete interactions. A Nunc™ OmniTray™ was prepared with 35 ml of fifth potato dextrose agar (PDA) with 10 plugs as inoculum (5mm) of either *R. solani* or *P. aphanidermatum*. *P*. *fluorescens* In5 cells were streaked 3 cm away from the plugs and incubated at 48 h at 20ºC.
