# Supplementary material for: Transcriptomic profiling of microbe–microbe interactions reveals the specific response of the biocontrol strain P. fluorescens In5 to the phytopathogen Rhizoctonia solani
Source: BMC Res Notes. 2017 Aug 10;10:376. doi: 10.1186/s13104-017-2704-8 (PMC5557065; doi:10.1186/s13104-017-2704-8)
Supplement: Supplementary file 2 — Additional file 2. Gene expression matrix of RNA-seq data of P. fluorescens In5 during interactions with R. solani and P. aphanidermatum. Graph represents 2log of expression means for each treatment (R. solani, Rs or P. aphanidermatum, Pa) compared to the control (C). [file 13104_2017_2704_MOESM2_ESM.docx]

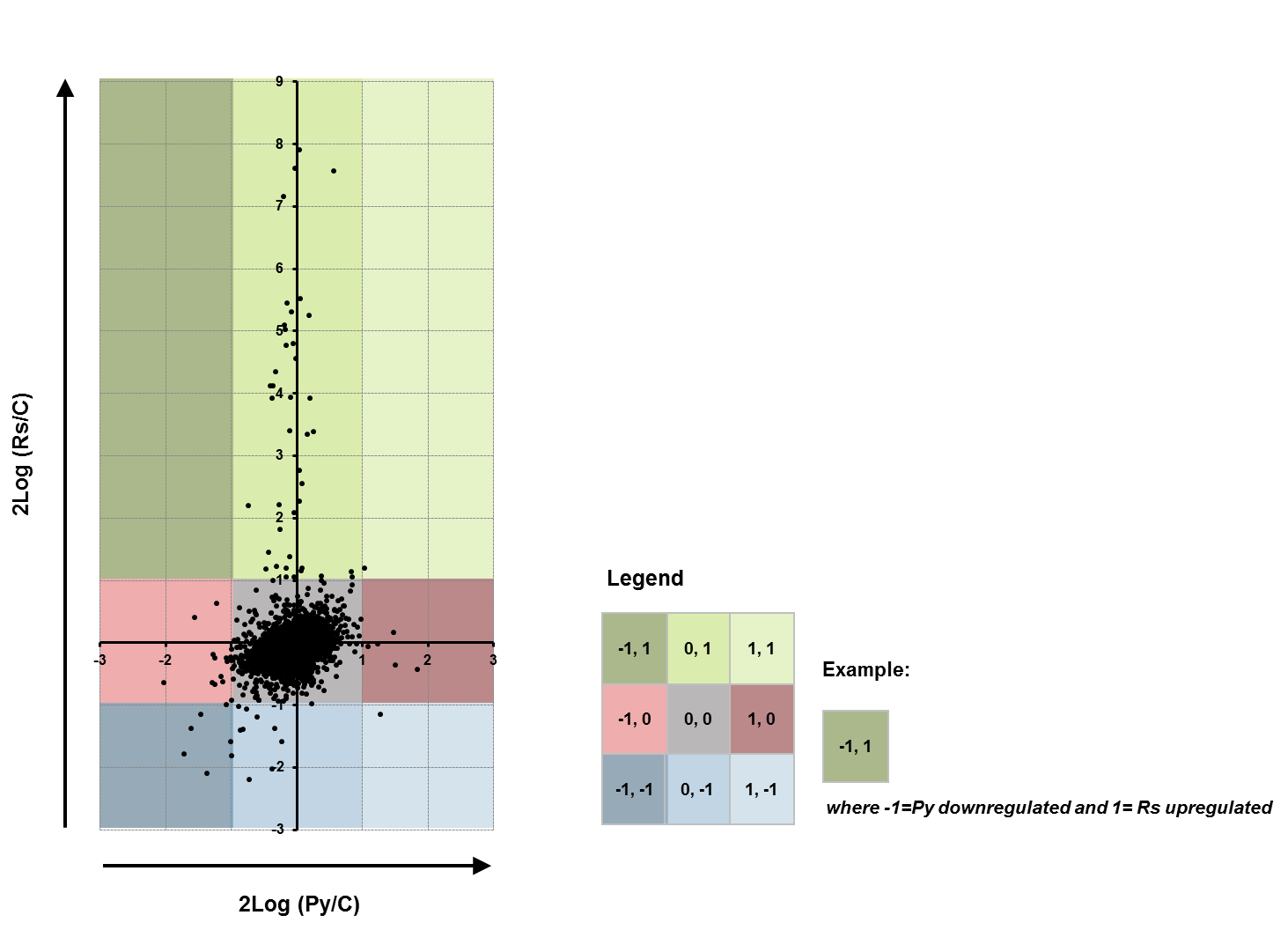


**Additional File 2. Gene expression matrix of RNA-seq data of *P*. *fluorescens* In5 during interactions with *R. solani* and *P. aphanidermatum*.** Graph represents 2log of expression means for each treatment (*R. solani*, Rs or *P. aphanidermatum*, Py) compared to the control (C).
