# Supplementary material for: Transcriptomic profiling of microbe–microbe interactions reveals the specific response of the biocontrol strain P. fluorescens In5 to the phytopathogen Rhizoctonia solani
Source: BMC Res Notes. 2017 Aug 10;10:376. doi: 10.1186/s13104-017-2704-8 (PMC5557065; doi:10.1186/s13104-017-2704-8)
Supplement: Supplementary file 3 — Additional file 3: Table S1. Significance testing of transcriptomic data. Transcripts significantly (P<0.05) up- (↑) or downregulated (↓) from the control (Pseudomonas fluorescens In5) in dual-culture with Rhizoctonia solani (Rs) compared to Pythium aphanidermatum (Pa) are indicated by 1 (red box) whereas transcripts not significantly (P>0.05) differentially expressed from control are represented as 0 (green box). Only genes up-or downregulated three-fold were included. [file 13104_2017_2704_MOESM3_ESM.docx]

**Table S1 Significance testing of transcriptomic data**. Transcripts significantly (*P*<0.05) up- (↑) or downregulated (↓) from the control (*Pseudomonas fluorescens* In5) in dual-culture with *Rhizoctonia solani* (Rs) compared to *Pythium aphanidermatum* (Pa) are indicated by1 (grey) whereas transcripts not significantly (*P*>0.05) differentially expressed from control are represented as 0 (white). Only genes up-or downregulated three-fold were included.

| **Locus Tag** | **Pa↑** | **Pa↓** | **Rs↑** | **Rs↓** | **Rs↑ Pa↑** | **Rs↓ Pa↓** | **GenBank ID** | **Protein Name** |
| --- | --- | --- | --- | --- | --- | --- | --- | --- |
| AL066_06105 | **0** | **0** | **0** | **0** | **0** | **0** | KPN89919.1 | alpha/beta hydrolase |
| AL066_09895 | **0** | **0** | **1** | **0** | **0** | **0** | KPN90627.1 | hydrolase |
| AL066_14420 | **0** | **0** | **0** | **0** | **0** | **0** | KPN91473.1 | quercetin 2,3-dioxygenase |
| AL066_18305 | **0** | **0** | **1** | **0** | **0** | **0** | KPN92189.1 | FMN-dependent NADH-azoreductase |
| AL066_05010 | **0** | **0** | **1** | **0** | **0** | **0** | KPN94213.1 | hypothetical protein |
| AL066_10105 | **0** | **0** | **1** | **0** | **0** | **0** | KPN90667.1 | pirin |
| AL066_04230 | **0** | **0** | **1** | **0** | **0** | **0** | KPN94078.1 | aromatic ring-opening dioxygenase LigB |
| AL066_06100 | **0** | **0** | **0** | **0** | **0** | **0** | KPN89918.1 | mechanosensitive ion channel protein MscS |
| AL066_31290 | **0** | **1** | **1** | **0** | **0** | **0** | KPN87407.1 | hypothetical protein |
| AL066_13550 | **0** | **1** | **1** | **0** | **0** | **0** | KPN91317.1 | phage infection protein |
| AL066_14055 | **0** | **0** | **1** | **0** | **0** | **0** | KPN91408.1 | hypothetical protein |
| AL066_13570 | **0** | **1** | **1** | **0** | **0** | **0** | KPN91319.1 | hypothetical protein |
| AL066_05530 | **0** | **0** | **1** | **0** | **0** | **0** | KPN89818.1 | glutathionyl-hydroquinone reductase YqjG |
| AL066_06700 | **0** | **0** | **1** | **0** | **0** | **0** | KPN90033.1 | hypothetical protein |
| AL066_06705 | **0** | **1** | **1** | **0** | **0** | **0** | KPN90034.1 | hypothetical protein |
| AL066_12150 | **0** | **1** | **0** | **0** | **0** | **0** | KPN91047.1 | hypothetical protein |
| AL066_12145 | **0** | **0** | **0** | **0** | **0** | **0** | KPN91046.1 | hypothetical protein |
| AL066_07630 | **0** | **1** | **1** | **0** | **0** | **0** | KPN90211.1 | hypothetical protein |
| AL066_07090 | **1** | **0** | **1** | **0** | **1** | **0** | KPN90105.1 | ATPase |
| AL066_13695 | **0** | **0** | **0** | **0** | **0** | **0** | KPN91341.1 | hypothetical protein |
| AL066_31095 | **0** | **0** | **1** | **0** | **0** | **0** | KPN87516.1 | filamentous hemagglutinin |
| AL066_10590 | **1** | **0** | **1** | **0** | **1** | **0** | KPN90754.1 | FMN-dependent NADH-azoreductase |
| AL066_31575 | **0** | **0** | **1** | **0** | **0** | **0** | KPN87458.1 | quercetin 2,3-dioxygenase |
| AL066_04065 | **0** | **0** | **1** | **0** | **0** | **0** | KPN94047.1 | DoxX family protein |
| AL066_07095 | **0** | **0** | **1** | **0** | **0** | **0** | KPN90106.1 | histidine kinase |
| **Locus Tag** | **Pa↑** | **Pa↓** | **Rs↑** | **Rs↓** | **Rs↑ Pa↑** | **Rs↓ Pa↓** | **GenBank ID** | **Protein Name** |
| AL066_22770 | **0** | **1** | **1** | **0** | **0** | **0** | KPN88932.1 | hypothetical protein |
| AL066_12155 | **0** | **1** | **1** | **0** | **0** | **0** | KPN91048.1 | hypothetical protein |
| AL066_03355 | **0** | **0** | **1** | **0** | **0** | **0** | KPN93911.1 | ABC transporter |
| AL066_27880 | **0** | **1** | **1** | **0** | **0** | **0** | KPN88055.1 | cytochrome b |
| AL066_11190 | **0** | **1** | **0** | **1** | **0** | **1** | KPN90872.1 | oxidoreductase |
| AL066_11490 | **0** | **0** | **0** | **1** | **0** | **0** | KPN90923.1 | hypothetical protein |
| AL066_26360 | **0** | **1** | **0** | **1** | **0** | **1** | KPN87765.1 | AlpA family transcriptional regulator |
| AL066_11195 | **0** | **0** | **0** | **1** | **0** | **0** | KPN90873.1 | sulfite reductase |
| AL066_13155 | **0** | **0** | **0** | **1** | **0** | **0** | KPN91243.1 | NIPSNAP domain containing protein |
| AL066_11200 | **0** | **1** | **0** | **1** | **0** | **1** | KPN92978.1 | cytochrome C oxidase Cbb3 |
| AL066_11050 | **0** | **1** | **0** | **1** | **0** | **1** | KPN90845.1 | (Fe-S)-binding protein |
