# Supplementary material for: Transcriptomic profiling of microbe–microbe interactions reveals the specific response of the biocontrol strain P. fluorescens In5 to the phytopathogen Rhizoctonia solani
Source: BMC Res Notes. 2017 Aug 10;10:376. doi: 10.1186/s13104-017-2704-8 (PMC5557065; doi:10.1186/s13104-017-2704-8)
Supplement: Supplementary file 4 — Additional file 4: Table S2. Significance testing of transcriptomic data. Transcripts significantly (P<0.05) up- (↑) or downregulated (↓) from the control (Pseudomonas fluorescens In5) in dual-culture with Pythium aphanidermatum (Pa) compared to Rhizoctonia solani (Rs) are indicated by 1 (red box) whereas transcripts not significantly (P>0.05) differentially expressed from control are represented as 0 (green box). Only genes up-or downregulated two-fold were included. [file 13104_2017_2704_MOESM4_ESM.docx]

**Table S2 Significance testing of transcriptomic data**. Transcripts significantly (*P*<0.05) up- (↑) or downregulated (↓) from the control (*Pseudomonas fluorescens* In5) in dual-culture with *Pythium aphanidermatum* (Pa) compared to *Rhizoctonia solani* (Rs) are indicated by 1 (grey) whereas transcripts not significantly (*P*>0.05) differentially expressed from control are represented as 0 (white). Only genes up-or downregulated two-fold were included.

| **Locus Tag** | **Pa↑** | **Pa↓** | **Rs↑** | **Rs↓** | **Rs↑ Pa↑** | **Rs↓ Pa↓** | **GenBank ID** | **Protein Name** |
| --- | --- | --- | --- | --- | --- | --- | --- | --- |
| AL066_27440 | **1** | **0** | **0** | **0** | **0** | **0** | KPN87969.1 | hypothetical protein |
| AL066_27625 | **1** | **0** | **0** | **0** | **0** | **0** | KPN88005.1 | glycine/betaine ABC transporter substrate-binding protein |
| AL066_02610 | **1** | **0** | **0** | **0** | **0** | **0** | KPN93775.1 | hypothetical protein |
| AL066_27960 | **1** | **0** | **0** | **0** | **0** | **0** | KPN88071.1 | malonate decarboxylase subunit delta |
| AL066_25950 | **0** | **0** | **0** | **0** | **0** | **0** | KPN87688.1 | hypothetical protein |
| AL066_07605 | **1** | **0** | **0** | **0** | **0** | **0** | KPN90206.1 | ABC transporter permease |
| AL066_04890 | **0** | **0** | **0** | **0** | **0** | **0** | KPN94191.1 | antibiotic synthesis protein MbtH |
| AL066_08545 | **0** | **1** | **0** | **0** | **0** | **0** | KPN90381.1 | PseC |
| AL066_05775 | **0** | **1** | **0** | **0** | **0** | **0** | KPN89865.1 | hypothetical protein |
| AL066_27365 | **0** | **1** | **0** | **1** | **0** | **1** | KPN87956.1 | biotin synthase |
| AL066_11260 | **0** | **1** | **0** | **1** | **0** | **1** | KPN90883.1 | cytochrome C |
| AL066_07745 | **0** | **1** | **0** | **0** | **0** | **0** | KPN90233.1 | beta-lactamase |
| AL066_17410 | **0** | **1** | **0** | **0** | **0** | **0** | KPN93038.1 | hypothetical protein |
| AL066_14095 | **0** | **1** | **0** | **1** | **0** | **1** | KPN91415.1 | hypothetical protein |
| AL066_23365 | **0** | **1** | **0** | **0** | **0** | **0** | KPN89047.1 | hypothetical protein |
| AL066_17940 | **0** | **1** | **0** | **1** | **0** | **1** | KPN92120.1 | phenylalanine 4-monooxygenase |
| AL066_10185 | **0** | **1** | **0** | **0** | **0** | **0** | KPN90680.1 | hypothetical protein |
| AL066_11200 | **0** | **1** | **0** | **1** | **0** | **1** | KPN92978.1 | cytochrome C oxidase Cbb3 |
| AL066_15755 | **0** | **1** | **0** | **1** | **0** | **1** | KPN91711.1 | terminase |
| AL066_15780 | **0** | **1** | **0** | **0** | **0** | **0** | KPN91716.1 | transcriptional regulator |
| AL066_24330 | **0** | **1** | **0** | **1** | **0** | **1** | KPN89229.1 | hypothetical protein |
| AL066_26360 | **0** | **1** | **0** | **1** | **0** | **1** | KPN87765.1 | AlpA family transcriptional regulator |
| AL066_11985 | **0** | **1** | **0** | **0** | **0** | **0** | KPN91015.1 | hypothetical protein |
