# Supplementary material for: Transcriptomic profiling of microbe–microbe interactions reveals the specific response of the biocontrol strain P. fluorescens In5 to the phytopathogen Rhizoctonia solani
Source: BMC Res Notes. 2017 Aug 10;10:376. doi: 10.1186/s13104-017-2704-8 (PMC5557065; doi:10.1186/s13104-017-2704-8)
Supplement: Supplementary file 5 — Additional file 5. Characteristics of the hypothetical proteins encoded by genes upregulated in response to R. solani. [file 13104_2017_2704_MOESM5_ESM.docx]

**Additional File 5. Characteristics of the hypothetical proteins encoded by genes upregulated in response to *R. solani*.**

| **ID** | **SignalP*^a^*** |  |  | **SecretomeP*^b^*** | **Phobius*^c^*** |  |  |  |  | **AMPD*^d^*** |  |  |  |
| --- | --- | --- | --- | --- | --- | --- | --- | --- | --- | --- | --- | --- | --- |
| AL066_ | Secreted | P(0.5) | Cleavage Site | P(0.5) | Secreted | Cyt | TM | Out | AA | Charge | Cys | Ahelix | PossAMP |
| 5010 | Yes | 0.675 | 17 |  |  |  |  |  | 72 | -2 | No | Amph | Yes |
| 31290 | Yes | 0.806 | 20 |  |  |  |  |  | 112 | -2 | No | Amph | Yes |
| 14055 | Yes | 0.763 | 20 |  |  |  |  |  | 91 | 3 | Yes |  | Yes |
| 13570 | Yes | 0.855 | 20 |  |  |  |  |  | 99 | -4 | No | Amph | Yes |
| 6700 | Yes | 0.72 | 20 |  |  |  |  |  | 64 | 2 | No | Amph | Yes |
| 6705 | No | 0.33 |  | 0.056 | Yes |  |  | 15-71 | 71 | -4 | No | Amph | Yes |
| 12150 | No | 0.293 |  | 0.297 | No | 1-11 | 12-31 | 32-70 | 70 |  |  |  | No |
| 12145 | No | 0.373 | 34 | 0.102 | Yes |  |  | 34-51 | 51 | 1 | No | Amph | Yes |
| 7630 | Yes | 0.903 | 21 |  |  |  |  |  | 60 | 1 | No | Amph | Yes |
| 13695 | Yes | 0.871 | 20 |  |  |  |  |  | 113 | -13 | No | Amph | Yes |
| 22770 | Yes | 0.89 | 20 |  |  |  |  |  | 150 | -9 | No |  | No |
| 12155 | Yes | 0.695 | 24 |  |  |  |  |  | 35 | 2 | No | Amph | Yes |

*^a^*SignalP, (<http://www.cbs.dtu.dk/services/SignalP/>); *^b^*SecretomeP, (<http://www.cbs.dtu.dk/services/SecretomeP/>); *^c^*Phobius, (<http://phobius.sbc.su.se/>); *^d^*AMPD; antimicrobial peptide database, only secreted (http://aps.unmc.edu/AP/main.php)
